# Clinical target volume delineation including elective nodal irradiation in preoperative and definitive radiotherapy of pancreatic cancer

**DOI:** 10.1186/1748-717X-7-86

**Published:** 2012-06-12

**Authors:** Luciana Caravatta, Giuseppina Sallustio, Fabio Pacelli, Gilbert DA Padula, Francesco Deodato, Gabriella Macchia, Mariangela Massaccesi, Vincenzo Picardi, Savino Cilla, Alfonso Marinelli, Numa Cellini, Vincenzo Valentini, Alessio G Morganti

**Affiliations:** 1 Radiotherapy Unit, Department of Oncology, Fondazione di Ricerca e Cura “Giovanni Paolo II”, Università Cattolica del S. Cuore, Largo A. Gemelli 1, 86100, Campobasso, Italy; 2 Radiology Unit, Fondazione di Ricerca e Cura “Giovanni Paolo II”, Università Cattolica del S. Cuore, Largo A. Gemelli 1, 86100, Campobasso, Italy; 3 Surgery Unit, Department of Oncology, Fondazione di Ricerca e Cura “Giovanni Paolo II”, Università Cattolica del S. Cuore, Largo A. Gemelli 1, 86100, Campobasso, Italy; 4 Radiation Oncology Department, The Lacks Cancer Center Saint Mary’s Health Care, Grand Rapids, MI, USA; 5 Physics Unit, Fondazione di Ricerca e Cura Giovanni Paolo II, Università Cattolica del S. Cuore, Campobasso, Italy; 6 Radiotherapy Department, Università Cattolica del S. Cuore, Rome, Italy

**Keywords:** Radiotherapy, Pancreatic cancer, Clinical target volume

## Abstract

**Background:**

Radiotherapy (RT) is widely used in the treatment of pancreatic cancer. Currently, recommendation has been given for the delineation of the clinical target volume (CTV) in adjuvant RT. Based on recently reviewed pathologic data, the aim of this study is to propose criteria for the CTV definition and delineation including elective nodal irradiation (ENI) in the preoperative and definitive treatment of pancreatic cancer.

**Methods:**

The anatomical structures of interest, as well as the abdominal vasculature were identified on intravenous contrast-enhanced CT scans of two different patients with pancreatic cancer of the head and the body. To delineate the lymph node area, a margin of 10 mm was added to the arteries.

**Results:**

We proposed a set of guidelines for elective treatment of high-risk nodal areas and CTV delineation. Reference CT images were provided.

**Conclusions:**

The proposed guidelines could be used for preoperative or definitive RT for carcinoma of the head and body of the pancreas. Further clinical investigations are needed to validate the defined CTVs.

## Background

Surgery is the only potentially curative modality for patients with clinically localized and operable pancreatic cancer [1]. Combined modality therapy compared to surgery alone has shown an advantage in terms of overall survival and should be considered for adjuvant treatment of resectable cancer patients [2-5]. Chemo-radiation represents a treatment option for patients with unresectable disease [6].

In particular, in our previous systematic review, an improvement of surgical resectability and in overall survival has been shown for patients with unresectable tumor treated with neoadjuvant chemo-radiotherapy and surgical resection (median survival: 16 – 32 months) [7]. Given the 5-year survival results (18-41%; median: 36%) in that publication on patients treated with preoperative radiotherapy (RT) [7], patients with unresectable pancreatic cancer without disease progression after chemo-radiotherapy should be considered for radical surgery and could be regarded as potentially curable.

Although there is no consensus concerning the elective nodal irradiation (ENI) in pancreatic cancer RT [8], it could be justified in a treatment with curative intent. Moreover, a high frequency of lymphatic spread (60–80%) was reported in head pancreatic cancer [9,10] and a high rate of local and nodal failure was noted in pathologic and clinical analyses (up to 75%) [11-13]. Based on these data, the prognosis of these patients could be theoretically improved reaching a higher local control and reducing the nodal recurrence rate [11-13], as already shown in resectable pancreatic carcinoma treated with ENI and concurrent chemotherapy (local recurrence rate with or without ENI: 0-13% vs 25%, respectively) [14,15].

Nevertheless, the close presence of organs at risk (OARs) such as kidneys, liver, small bowel, stomach, duodenum and spinal cord remain the main problems of abdominal radiotherapy, especially when large volume is treated. Therefore, CT-based definition of the clinical target volume (CTV) and 3D treatment planning (3D-CRT), thus reducing the dose to OARs, is strongly recommended and is currently considered the standard approach [8,16].

Further advantages can be achieved by intensity modulated radiotherapy (IMRT) [17], as well as by 4D treatment planning [18]. For both 3D and IMRT treatment planning, a proper knowledge, definition and delineation of CTV is required. Moreover, this issue became particularly relevant for IMRT-based treatment planning based on the dose gradients close to the planning target volume (PTV). For this reason, standardized contouring guidelines to ensure the adequacy of the CTV should be provided.

Few indications for the CTV definition of elective treatment in adjuvant or definitive radiotherapy have been given [19-22]. Generally, the treatment target volume delineation was related to the location of the primary disease and to the status of lymph node involvement [16]. Currently, no recommendations based on modern imaging modalities are available for preoperative or definitive RT.

Moreover, several anatomic and pathologic studies have been conducted to identify lymphatic network and high risk areas of lymph node involvement [23-27] and to define the pattern of perineural invasion of pancreatic cancer [27-29]. Concerning lymphatic drainage, a rich communication between the anterior surface of the head of the pancreas, the common hepatic artery, the celiac trunk origin and the superior mesenteric artery was described. As well as a lymphatic pathway from the body and the tail of pancreas was shown around the splenic blood vessels and the inferior pancreatic artery up to the lymph nodes situated on the left side of the celiac trunk and the superior mesenteric artery [23,24]. The extent of perineural invasion has been also demonstrated in a number of pathologic studies, often showing lymphatic emboli and neural invasions in the soft tissue adherent to the vessels and near to the metastatic nodes [27-29]. The close embryologic development relationship of lymphatic and nervous structures could justify the dual pathway of dissemination of pancreatic cancer along peripancreatic connective tissues [30].

In particular, a review of 18 pathologic reports (reported on 5954 resectable pancreatic cancer patients treated with radical surgery) was recently conducted to evaluate the probability of lymph node metastases and to define the high risk lymph nodal regions, related to the primary tumor site (head or body/tail of pancreas) [31]. Based on these reviewed pathologic data, the aim of this study is to propose criteria for CTV definition and delineation including ENI in the preoperative or exclusive treatment of pancreatic cancer.

## Methods

### Clinical target volume definition and delineation

Based on the review of Sun et al. [31], the high risk lymph node regions, related to the head and body/tail of the pancreas, were defined as ENI areas. Particularly, according to the reviewed data, each lymph nodal region with a probability of involvement ≥ 3% was considered to be at clinically significant risk and proposed as an ENI area.

All lymph nodal nomenclature is based on the General Rules for Cancer of the Pancreas published by the Japan Pancreas Society (JPS) [32]. Tables 1 and 2 show high risk lymph nodal regions for head and body/tail pancreatic carcinoma.

**Table 1:** High risk lymph node regions of the head pancreatic cancer

**Pancreatic head tumor**			
**Lymph node group**	**JPS Classification [**[32]**]**	**%**	Recommended margins *
Infrapyloric lymph nodes	Group 6	7.2	10 mm margin around the inferior border of the pylorus
Common hepatic artery lymph nodes	Group 8	9.8	10 mm margin around the common hepatic artery, from the origin of the artery (correspond to the superior border of the pancreas), on the anterior surface of the portal vein upper to the hilum of the liver
Celiac trunk lymph nodes	Group 9	3.7	10 mm margin around the celiac trunk
Hepatoduodenal ligament lymph nodes	Group 12	7.9	10 mm margin around the portal vein segment that runs anteromedial to the inferior vena cava and between the porta hepatis of the liver and the superior part of the duodenum
Posterior pancreaticoduodenal lymph nodes	Group 13	32.3	10 mm margin around the inferior - posterior pancreaticoduodenal artery
Superior mesenteric artery lymph nodes	Group 14	15.8	10 mm margin around the origin of superior mesenteric artery
Paraaortic lymph nodes	Group 16	10.9	10 mm margin around the abdominal aorta, between the celiac artery and the inferior mesenteric artery
Anterior pancreaticoduodenal lymph nodes	Group 17	19.8	10 mm margin around the superior - anterior pancreaticoduodenal artery

* also include any visible nodes (lower axis > 1 cm and/or FDG- avid on PET) plus a margin of 10 mm.

Lymph nodes nomenclature is based on the General Rules for Cancer of the Pancreas published by the Japan Pancreas Society (JPS). The anatomical structures of interest and the abdominal blood vessels of reference were identified for each lymph node region.

**Figure 1:**
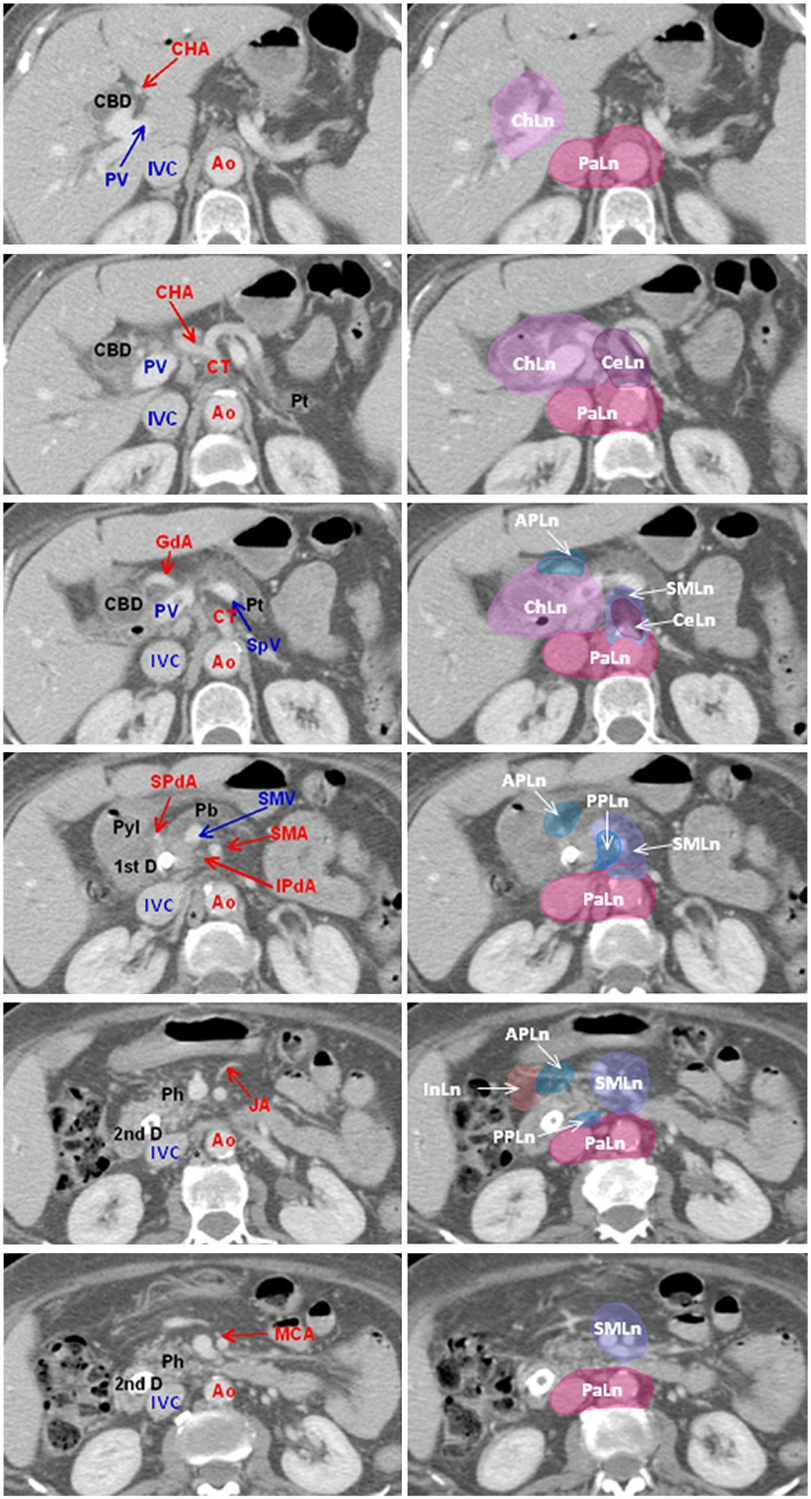
**Anatomical structures for the high risk lymph nodal regions of the head pancreatic cancer.** Transverse CT slices through high risk lymph node regions of the head pancreatic cancer from cranial to caudal direction. The abdominal artery and the anatomical structures were identified as a surrogate target for lymph node regions. A margin of 10 mm was added to the artery to delineate lymph node area, including the soft tissue with lymphatic and neural plexus. CTV was formed by the union of the identified lymph node areas. *Abbreviations*: Ao = Aorta; APLn = Anterior Pancreaticoduodenal Lymph nodes; CBD = Common Bile Duct; CeLn = Celiac Lymph nodes; CHA = Common Hepatic Artery; ChLn = Common hepatic and hepatoduodenal ligament Lymph nodes; CT = Celiac Trunk; 1st D = first part of Duodenum; GdA = Gastroduodenal Artery; InLn = Infrapyloric Llymph nodes; IPdA = Inferior Pancreaticoduodenal Artery; IVC = Inferior Vena Cava; JA = Jejunal Artery; MCA = Medial Colic Artery; PaLn = Paraaortic Lymph nodes; Pb = Pancreatic body; Ph = Pancreatic head; Pt = Pancreatic tail; Pyl = Pylorus; PPLn = Posterior Pancreaticoduodenal Lymph nodes; PV = Portal Vein; 2nd D = second part of Duodenum; SMA = Superior Mesenteric Artery; SMLn = Superior Mesenteric Lymph nodes; SMV = Superior Mesenteric Vein; SPdA = Superior Pancreaticoduodenal Artery; SpV = Splenic Vein; * = pillar of the diaphragm.

**Figure 2:**
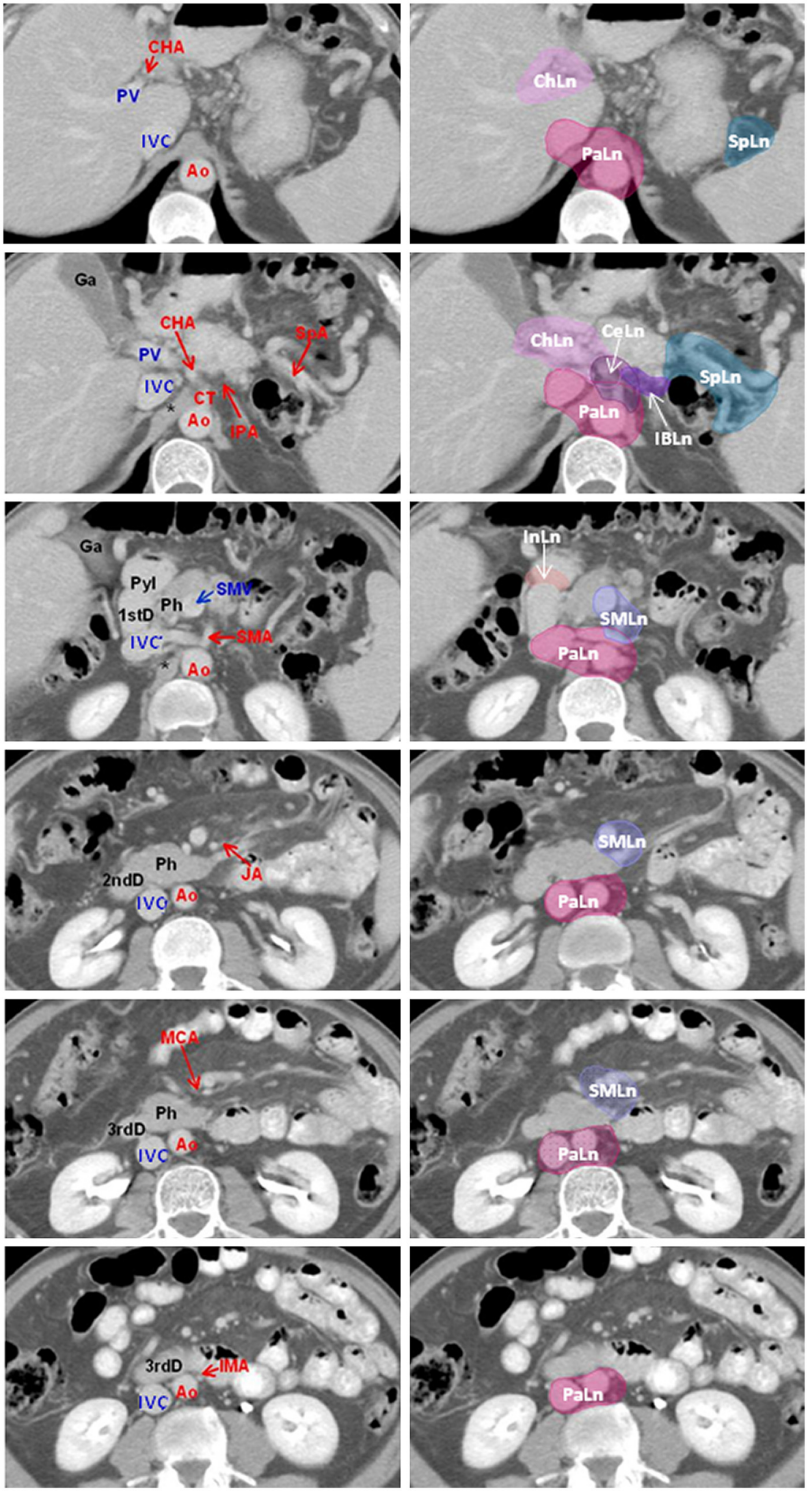
**Anatomical structures for the high risk lymph nodal regions of the body/tail pancreatic cancer.** Transverse CT slices through high risk lymph node regions of the body/tail pancreatic cancer from cranial to caudal direction. The abdominal artery and the anatomical structures were identified as a surrogate target for lymph node regions. A margin of 10 mm was added to the artery to delineate lymph node area, including the soft tissue with lymphatic and neural plexus. *Abbreviations*: Ao = Aorta; CeLn = Celiac Lymph nodes; CHA = Common Hepatic Artery; ChLn = Common hepatic and hepatoduodenal ligament Lymph nodes; CT = Celiac Trunk; 1st D = first part of Duodenum; Ga = Gallbladder; IBLn = Inferior Body Lymph nodes; IMA = Inferior Mesenteric Artery; InLn = Infrapyloric Lymph nodes; IPA = Inferior Pancreatic Artery; IVC = Inferior Vena Cava; JA = Jejunal Artery; MCA = Medial Colic Artery; PaLn = Paraaortic Lymph nodes; Ph = Pancreatic head; Pyl = Pylorus; PV = Portal Vein; 2nd D = second part of Duodenum; SpA = Splenic Artery; SpLn = hilus of the spleen and Splenic Lymph nodes; SMA = Superior Mesenteric Artery; SMLn = Superior Mesenteric Lymph nodes; SMV = Superior Mesenteric Vein; 3rd D = third part of Duodenum; * = pillar of the diaphragm.

Thin-cut dynamic multiphase helical CT scan of the was performed in two different patients for the delineation of CTV in the treatment of the head and the body pancreas adenocarcinoma, respectively. CT scans were performed with a high-speed scanner (CT Hi Speed Nx/i Pro; 2-slice; GE Medical System, Milwaukee,WI, USA) and were acquired with 3 mm thickness and 9 mms_1 table speed. Abdominal blood vessels and anatomical structures were identified as a surrogate region of interest for the delineation of high risk lymph nodal regions (Table 1). Correct localization of each anatomical structure was identified by the radiologist and the radiation oncologist by dynamic observation during all contrastographic phases (early arterial, arterial, portal and late phases) and delineated on late contrastographic phase. Based on pathologic studies [27-29], to delineate a lymph node area, a margin of 10 mm was added to the artery including the soft tissue with the lymphatic and neural plexus. This margin was not extended into the other normal tissue or structures, e.g. the vertebral body (Figures 1 and 2).

Other modifications were then made. In view of the proximal location of the hepatoduodenal ligament and the common hepatic artery, only one lymph node area (Group 8 and 12) was delineated for both structures (light violet area, Figures 1 and 2).

For lymph nodes around the superior mesenteric artery (Group 14), we included the soft tissue around the vessel based on the demonstrated subclinical metastatic rate [27-31]; because a moderate metastatic rate of the subgroup 14c (lymph nodes at the root of the medial colic artery) and the subgroup 14d (lymph nodes at the root of the jejunal artery) was observed [27-31], we suggested to include also the soft tissue around these vessels in the contouring (violet area, Figures 1 and 2).

Concerning the paraaortic lymph nodes (Group 16), pathologic reports [25,26,31,33] showed that the majority of positive lymph nodes was in the areas between the celiac artery and the inferior mesenteric artery, wherever the primary tumors were situated. Therefore, only this area was included in the contouring (red area, Figures 1 and 2). Moreover, in the area between the celiac artery and the inferior mesenteric artery, the positive lymph nodes were mainly located anterior to the abdominal aorta and between the abdominal aorta and the inferior vena cava. Therefore, the contouring was extended anteriorly, laterally and posteriorly to these vessels with the exception of the lateral side of the inferior vena cava (red area, Figures 1 and 2).

**Figure 3:**
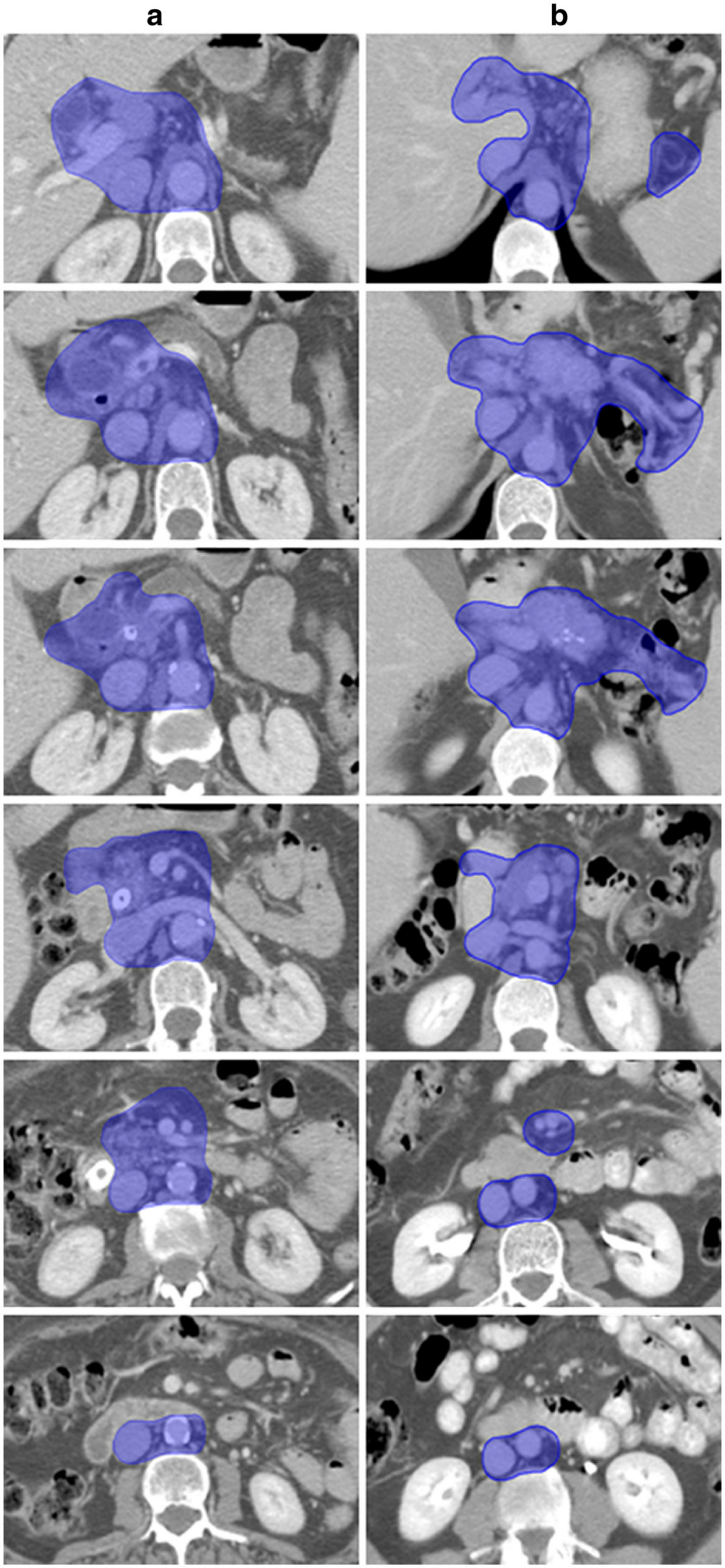
**CTV delineation in the treatment of the head and the body pancreas adenocarcinoma.** Transverse CT slices through high risk lymph node regions of the head (a) and body/tail (b) pancreatic cancer from cranial to caudal direction. CTV was formed by the union of the identified lymph node areas (blue area).

After all the ENI areas were delineated, the CTV was defined by the union of the identified areas as shown in Figure 3.

## Results

Two patients with unresectable pancreatic cancer of the head and body/tail were included in this study. Two different CTVs have been defined according to the reviewed data and the different tumor location in the head or body- tail of the pancreas [31]. The reference images, produced at 10 mm of distance between each slice, are shown for both tumor sites in Figure 3a and 3b, respectively.

For tumors located in the head of the pancreas, the CTV was defined by the union of the lymphatic areas around the infrapyloric region (Group 6), the common hepatic artery (Group 8), the celiac trunk (Group 9), the hepatoduodenal ligament (Group 12), the posterior pancreaticoduodenal artery (Group 13), the superior mesenteric artery (Group 14), the paraaortic area (Group 16) and the anterior pancreaticoduodenal artery (Group 17) (blue areas, Figure 3a).

In patients with pancreatic body- tail tumor lymph nodes around the common hepatic artery (Group 8), the celiac trunk (Group 9), the splenic artery and the ilus of spleen (Group 10 and 11), the hepatoduodenal ligament (Group 12), the superior mesenteric artery (Group 14), the paraaortic region (Group 16), the inferior body area (Group 18) were included in the CTV (blue areas, Figure 3b).

In both cases the primary tumor was obvious included into the CTV, with a 3 cm margin in the pancreatic tissue.

More details about the anatomical structures of interest and the abdominal blood vessels of reference for each lymph node region were described in Tables 1 and 2.

**Table 2:** High risk lymph node regions of the body/tail pancreatic cancer

**Pancreatic body/tail tumor**			
**Lymph node group**	**JPS Classification [**[32]**]**	**%**	Recommended margins *
Infrapyloric lymph nodes	Group 6	3.3	10 mm margin around the inferior border of the pylorus
Common hepatic artery lymph nodes	Group 8	15.1	10 mm margin around the common hepatic artery, from the origin of the artery (correspond to the superior border of the pancreas), on the anterior surface of the portal vein upper to the hilum of the liver
Celiac trunk lymph nodes	Group 9	9.6	10 mm margin around the celiac trunk
Hilus of the spleen lymph nodes	Group 10	4.1	10 mm margin around each visible splenic vessel
Splenic artery lymph nodes	Group 11	35.6	10 mm margin around the splenic artery
Hepatoduodenal ligament lymph nodes	Group 12	8.2	10 mm margin around the portal vein segment that runs anteromedial to the inferior vena cava and between the porta hepatis of the liver and the superior part of the duodenum
Superior mesenteric artery lymph nodes	Group 14	9.6	10 mm margin around the origin of superior mesenteric artery
Paraaortic lymph nodes	Group 16	16.4	10 mm margin around the abdominal aorta, between the celiac artery and the inferior mesenteric artery
Inferior body lymph nodes	Group 18	24.7	10 mm margin around the inferior pancreatic artery

* also include any visible nodes (lower axis > 1 cm and/or FDG- avid on PET) plus a margin of 10 mm.

Lymph nodes nomenclature is based on the General Rules for Cancer of the Pancreas published by the Japan Pancreas Society (JPS). The anatomical structures of interest and the abdominal blood vessels of reference were identified for each lymph node region.

## Discussion

The aim of this investigation was to propose standard criteria for the CTV definition and delineation in the preoperative or exclusive treatment of pancreatic cancer, with particular attention to elective lymph node areas.

It must be admitted that ENI is controversial and may be considered questionable [8]. In fact, palliative RT has been shown to be effective even without prophylactive nodal irradiation [34].

Furthermore, in order to increase the resectability, a dose escalation to the Gross Tumor Volume (GTV) more than a prophylactic dose may be required, as well as the inclusion of lymph nodes in the irradiated volume may be associated with increased toxicity and represent a limit for concurrent chemotherapy [35,36].

However, we must recognize that a high incidence of local and nodal failure was noted in pathologic and clinical analyses [11-13]. Based on these data, the prognosis of patients with pancreatic carcinoma remains conditioned by low local control and by a high nodal recurrence rate (21-47%) [11-13]. Moreover, the use of new technologies (IMRT, 4D-RT) could reduce the risk of toxicity related to ENI, and allow dose-escalation studies to improve the local control rate [17,18].

Guidelines for the delineation of ENI were proposed by Brunner et al. [19] and concerned only the treatment of head pancreatic carcinoma. Based on pathologic evaluation of 175 patients with ductal head pancreatic carcinoma who underwent radical pancreatoduodenectomy, the study confirmed the high probability of lymphatic spread and the need of the elective irradiation of regional and paraaortic lymphatic areas. Indeed, the total incidence of regional lymph node metastasis was 76% (133/175 cases) and the posterior pancreaticoduodenal area, superior and inferior pancreatic head margin, anterior pancreaticoduodenal area, hepatoduodenal ligament, superior pancreatic body and superior mesenteric artery were identified as high-risk lymphatic involvement areas and selected for elective treatment [19].

In our study, based on the recent pathologic data review from Sun et al. [31], criteria for the CTV definition and delineation were proposed. One potential limitation of our proposal could be that many anatomical data are derived from Japanese studies. Unfortunately, since the unavailability at moment of large series in Europe, we needed to refer to the most relevant published scientific data.

A cut-off value of 3% risk of lymph node involvement, as reported in Sun et al. [31], was used to identify lymph nodal regions to be included in the CTV. This cut-off value may seem relatively low. However, in the Sun’s study the lymph node metastatic incidence was pathologically evaluated in resectable low-stage carcinomas, while radiation therapy is often used in patients with advanced disease. Therefore, we considered the 3% cut-off value appropriate. Moreover, as is shown in the Tables 1 and 2, if a “classical” cut off of 10-15% was been chosen, some commonly considered high risk lymph node areas in post-operative setting (as common hepatic artery lymph nodes, hepatoduodenal ligament lymph nodes, celiac trunk lymph nodes, paraaortic lymph nodes for head tumors and hilus of the spleen lymph nodes for body/tail tumors) would been excluded.

A 10 mm margin was added around the arteries to define the lymphatic area, according to pathologic studies [27-29], where tumor infiltration was demonstrated in the soft tissue area with lymphatic and neural plexus, 10 mm around the artery. Furthermore, previous guidelines for pelvic lymph nodes delineation using IMRT, showed the possibility to cover 94% of nodes using a 10 mm margin around arteries [37].

Looking at these criteria, for tumors located in the head of the pancreas, we proposed the inclusion in the elective CTV of the following nodal areas: the infrapyloric lymph nodes (Group 6), the lymph nodes around the common hepatic (Group 8) and the hepatoduodenal ligament (Group 12), the celiac trunk lymph nodes (Group 9), the posterior pancreaticoduodenal lymph nodes (Group 13), the superior mesenteric lymph nodes (Group 14), the paraaortic lymph nodes (Group 16) and the anterior pancreaticoduodenal lymph nodes (Group 17). For patients with pancreatic body and tail disease, we included the lymph nodes around the common hepatic artery (Group 8), the celiac trunk (Group 9), the splenic artery and the ilus of spleen (Group 10 and 11), the hepatoduodenal ligament (Group 12), the superior mesenteric artery (Group 14), the paraaortic region (Group 16), and the inferior body area (Group 18).

Although these indications are quite comparable to that reported by Brunner et al. [19], concerning the treatment of the head pancreatic adenocarcinoma, they appear to be partly different from those commonly reported by the currently reference literature [8,38]. Particularly, lymph nodes around the common hepatic artery (Group 8) and the hepatoduodenal ligament (Group 12) were included in the CTV also for body- tail tumors.

In this study we proposed a method for CTV definition and delineation in the preoperative and definitive treatment of pancreatic cancer. Based on our institutional evaluation and a previous published study [39] a margin of 12 mm, 7 mm and 5 mm, in the craniocaudal, lateral and anterior, and posterior direction, were respectively considered appropriated from the CTV to the PTV. Moreover, given that no close assumption can been taken about comparison with standard technique in term of impact on OAR irradiation, by using this method we strongly recommend a careful evaluation of DVH, especially if concurrent Gemcitabine has been administered.

Furthermore, since a large inter-observer variance has been shown [40], if standardized anatomical structures of reference are defined for each lymph node regions and recognized in the individual patient, a more reproducible and a tailored patient volume may be obtained.

## Conclusions

Based on the incidence of lymph node metastases, we developed a proposal for target contouring in pancreatic cancer. This proposal may represent a basis for a multi-institutional consensus on contouring guidelines in these tumors. However, in order to validate these guidelines through patterns of failures studies, in our center a dose escalation study using a Volumetric-Modulated Arc Therapy (VMAT) technique with Simultaneous Integrated Boost (SIB) was designed. After the treatment, patients will be re-evaluated by FDG-PET/CT scan and CT scan with contrast every 3 months for 3 years and every 6 months in the following 2 years.

## Competing interests

All authors disclose any financial and personal relationships with other people or organizations that could inappropriately influence (bias) their work.

## Authors’ contributions

LC, FD, GM, MM, VP, SC, AGM carried out the data and drafted the manuscript. LC and AGM coordinated the entire study. LC and GS identified and delineated the correct localization of each anatomical structure on the diagnostic images. LC and AM provided the pictures elaboration. FP, GDAP, NC, and VV critically revised the study and the manuscript. AGM provided the conception of this study and the final approval of the version to be published. All authors read and approved the final manuscript.

Presented in part at the XX Congress of Italian Radiotherapy Association, Naples, Italy, November 13–16, 2010.
